# Supplementary material for: A tissue-intrinsic mechanism sensitizes HIV-1 particles for TLR-triggered innate immune responses
Source: Nat Commun. 2026 May 9;17:4209. doi: 10.1038/s41467-026-72586-3 (PMC13157497; doi:10.1038/s41467-026-72586-3)
Supplement: Supplementary file 1 — Supplementary Information [file 41467_2026_72586_MOESM1_ESM.pdf]

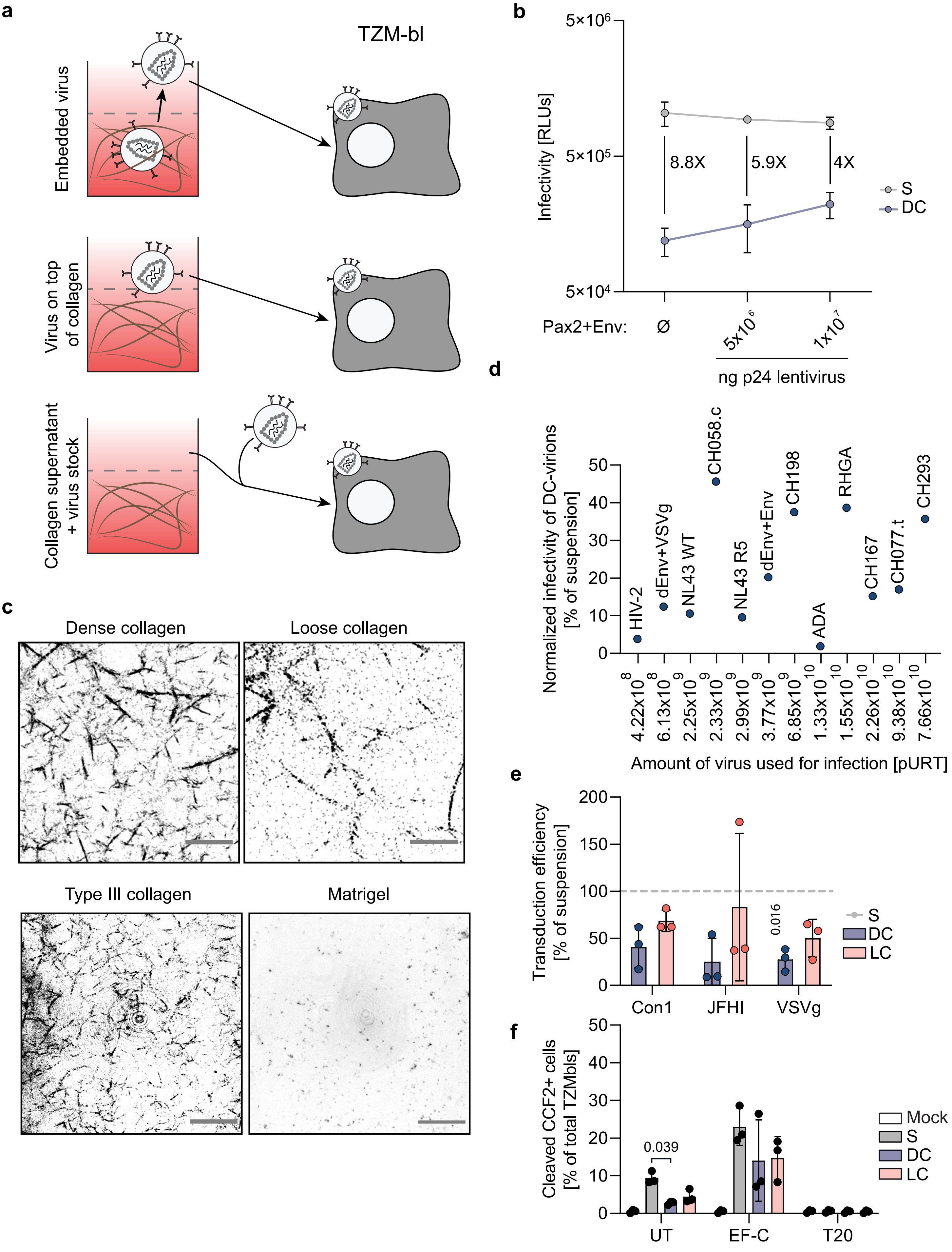

a
TZM-bl
Embedded virus
Virus on top of collagen
Collagen supernatant + virus stock
b
Infectivity [RLUs]
5×10⁶
5×10⁵
5×10⁴
8.8X
5.9X
4X
S
DC
Pax2+Env:
Ø
5x10⁶
1x10⁷
ng p24 lentivirus
c
Dense collagen
Loose collagen
Type III collagen
Matrigel
d
Normalized infectivity of DC-virions [% of suspension]
HIV-2
dEnv+VSVg
NL43 WT
CH058.c
NL43 R5
dEnv+Env
CH198
ADA
RHGA
CH167
CH077.t
CH293
4.22x10⁸
6.13x10⁸
2.25x10⁹
2.33x10⁹
2.99x10⁹
3.77x10⁹
6.85x10⁹
1.33x10¹⁰
1.55x10¹⁰
2.26x10¹⁰
9.38x10¹⁰
7.66x10¹⁰
Amount of virus used for infection [pURT]
e
Transduction efficiency [% of suspension]
0.016
S
DC
LC
Con1
JFHI
VSVg
f
Cleaved CCF2+ cells [% of total TZMbls]
0.039
Mock
S
DC
LC
UT
EF-C
T20

Supplementary Figure 1: Adhesive matrices exert a restriction on the infectivity of lentiviruses pseudotyped with different viral glycoproteins. **a**. Experimental workflow. Virions were either embedded in collagen matrices, seeded on top of already polymerized collagen gels, or combined with supernatants harvested from collagen matrices polymerized in the absence of virus. The supernatants were then used to infect TZM-bl cells for relative infectivity determination. **b**. Infectivity of HIV-1 NL4.3 virus after competition with lentiviral particles. HIV-1 virions were co-incubated in S or 2D DC in presence or absence of increasing amounts of lentiviral particles to compete for collagen binding. Equivalent amounts of HIV-1 RT units virions were then used for infection of TZM-bl reporter cells and infectivity determination. **c**. Representative micrographs. Different matrices were polymerized and imaged by confocal autoreflection microscopy. **d**. Correlation between the amounts of pURT used for infection for each viral strain and the extent of infectivity restriction after culture in DC gels. **e**. Transduction efficiency of Huh7.5 cells with lentiviral particles pseudotyped with the HCV glycoproteins Con1 or JFHI, or with VSVg after culture in S or DC. **f**. Quantification of the percentage of CCF2-product positive cells measured by flow cytometry. TZM-bl cells were infected with Vpr.Blam containing HIV-1 particles harvested from S, DC or LC cultures in presence or absence of the infectivity enhancer EF-C. Results represent the mean ± SD from 3 independent donors (**d,e**). Significance is indicated by p-values, and was calculated by two-way ANOVA; Tukey's post-test. Source data are provided as a Source Data file.

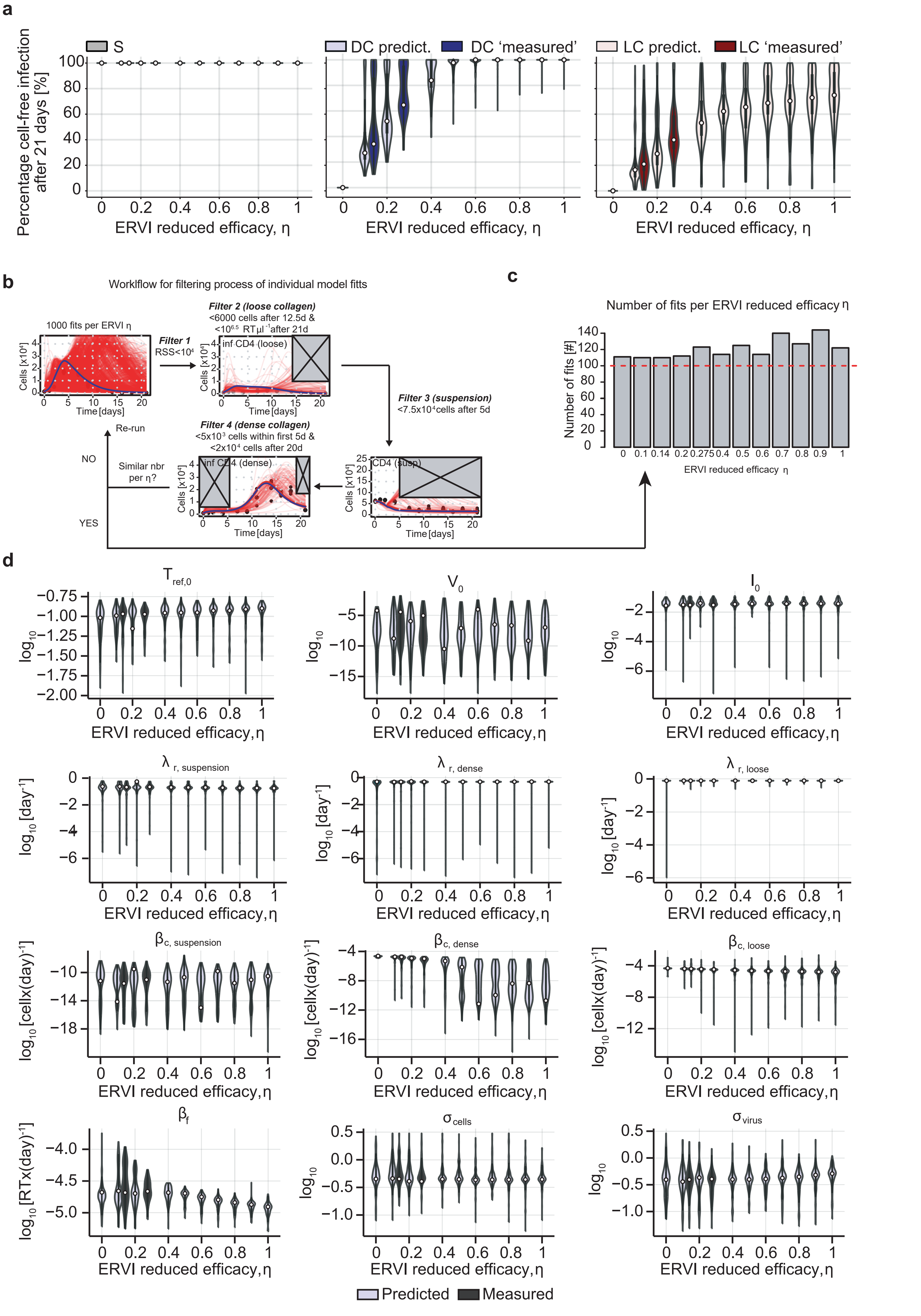
a
S
DC predict.
DC 'measured'
LC predict.
LC 'measured'
Percentage cell-free infection after 21 days [%]
ERVI reduced efficacy, η
b
Workflow for filtering process of individual model fits
1000 fits per ERVI η
Filter 1
RSS<10⁴
Filter 2 (loose collagen)
<6000 cells after 12.5d &
<10^6.5 RTμl⁻¹after 21d
inf CD4 (loose)
Filter 3 (suspension)
<7.5x10⁴cells after 5d
Filter 4 (dense collagen)
<5x10³ cells within first 5d &
<2x10⁴ cells after 20d
inf CD4 (dense)
CD4 (susp)
Cells [x10⁴]
Time [days]
Re-run
NO
Similar nbr per η?
YES
c
Number of fits per ERVI reduced efficacy η
Number of fits [#]
ERVI reduced efficacy η
d
T ref,0
V 0
I 0
λ r, suspension
λ r, dense
λ r, loose
β c, suspension
β c, dense
β c, loose
β f
σ cells
σ virus
log10
log10 [day⁻¹]
log10 [cellx(day)⁻¹]
log10 [RTx(day)⁻¹]
ERVI reduced efficacy, η
Predicted
Measured

Supplementary Figure 2: Modelling the consequences of ERVI for HIV-1 spread. a. Estimated fraction of primary CD4+ T cells infected by cell-free transmission after 21 days for the three environmental conditions and using the mathematical model explained in detail in (7) given different values for the assumed reduced efficacy of virion infectivity by ERVI, η, reanalyzing the data used in 7. The plots show the posterior distributions of estimates over ~110140 fits per value of η with dots indicating the estimate of the best model fit (see also Materials & Methods). Results for measured values of η = 0.14 (7) and η = 0.275 (here) are shown in dark colors. **b**. Filtering process of individual model fits to ensure comparability of estimates for different reduced efficacies η, used within the mathematical model shown in (7). A sequence of different filtering steps is applied to the results of the ensemble fits given different starting conditions only considering fits with residual sum of squares (RSS) <104 (filter 1), disregarding fits with predictions of >6000 cells after 12.5 days or viral concentrations > 106.8 RT • l-1 after 21 days in the supernatant for LC (filter 2), disregarding fits with >7.5×104 CD4 T cells after 5 days in suspension (filter 3), and excluding fits with >5000 CD4 T cells within the first 5 days or >2×104 cells after 20 days for DC. The procedure is repeated to ensure comparable number of estimates for each value of η after the filtering for the analysis. **c**. Number of fits per value of η considered within the analyses. d. Distribution of estimates for the individual parameters describing cell infection dynamics within suspension and 3D collagen environments. Results of the best fit for each value of η are indicated by the white dot. For the meaning of the individual parameters and specific model equations see Imle et al., 2019. Source data are provided as a Source Data file.

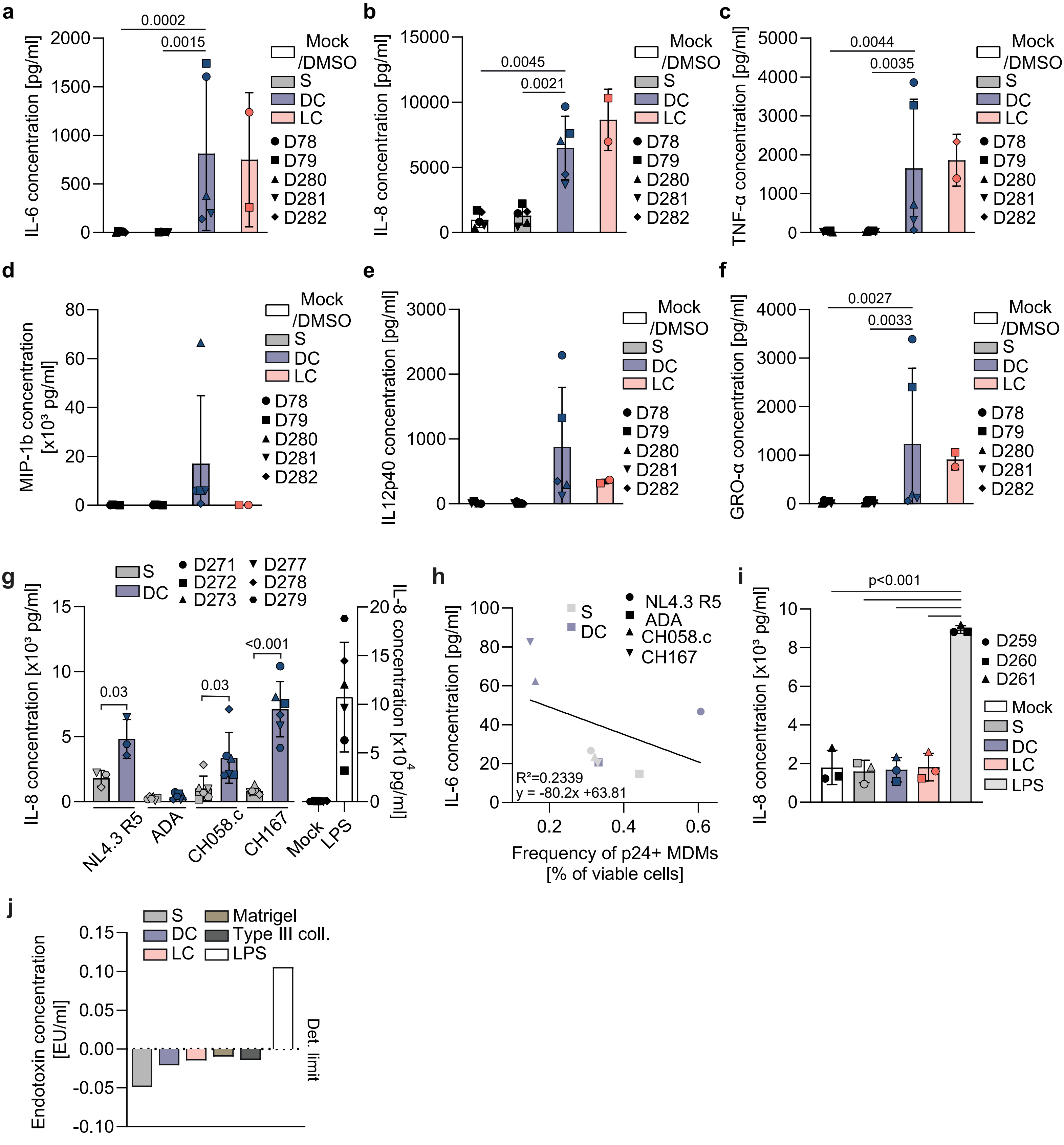

Supplementary Figure 3: The collagen induced immune sensitization of HIV-1 virions is not caused by soluble components or endotoxins. **a-f**. Quantification of individual cytokines from the analysis shown in Fig. 4 (e): IL-6 (**a**), IL-8 (**b**), TNF (**c**), MIP1β (**d**), IL12p40 (**e**) and GRO-α (**f**). **g**. Measurement of IL-8 release by ELISA form MDM supernatants. MDMs were cultured for 72h with S or DC derived HIV-1 strains virions prior to ELISA analysis. **h**. Correlation plot between MDMs infection rates and IL-6 secretion. Linear regression between the two parameters was performed. **i**. Measurement of IL-8 release by ELISA form MDM supernatants. MDMs were cultured for 72h with supernatants collected from S, DC or LC cultures without HIV-1 virus prior to ELISA analysis. **j**. Endotoxin measurement. The supernatants of S, type I DC/ LC or type III collagen, as well as matrigel matrices polymerized in the absence of virus were harvested, and the levels of endotoxin were determined. Results represent the mean ± SD from one experiment (**j**) or 3-5 independent donors (**a-i**). Significance is indicated by p-values, and was calculated by one-way ANOVA; Tukey's post-test (**i**), ratio-paired t-tests (**a**,**b**,**c**,**f**) and Wilcoxon matched-pairs signed rank test (**d**,**e**). Source data are provided as a Source Data file.

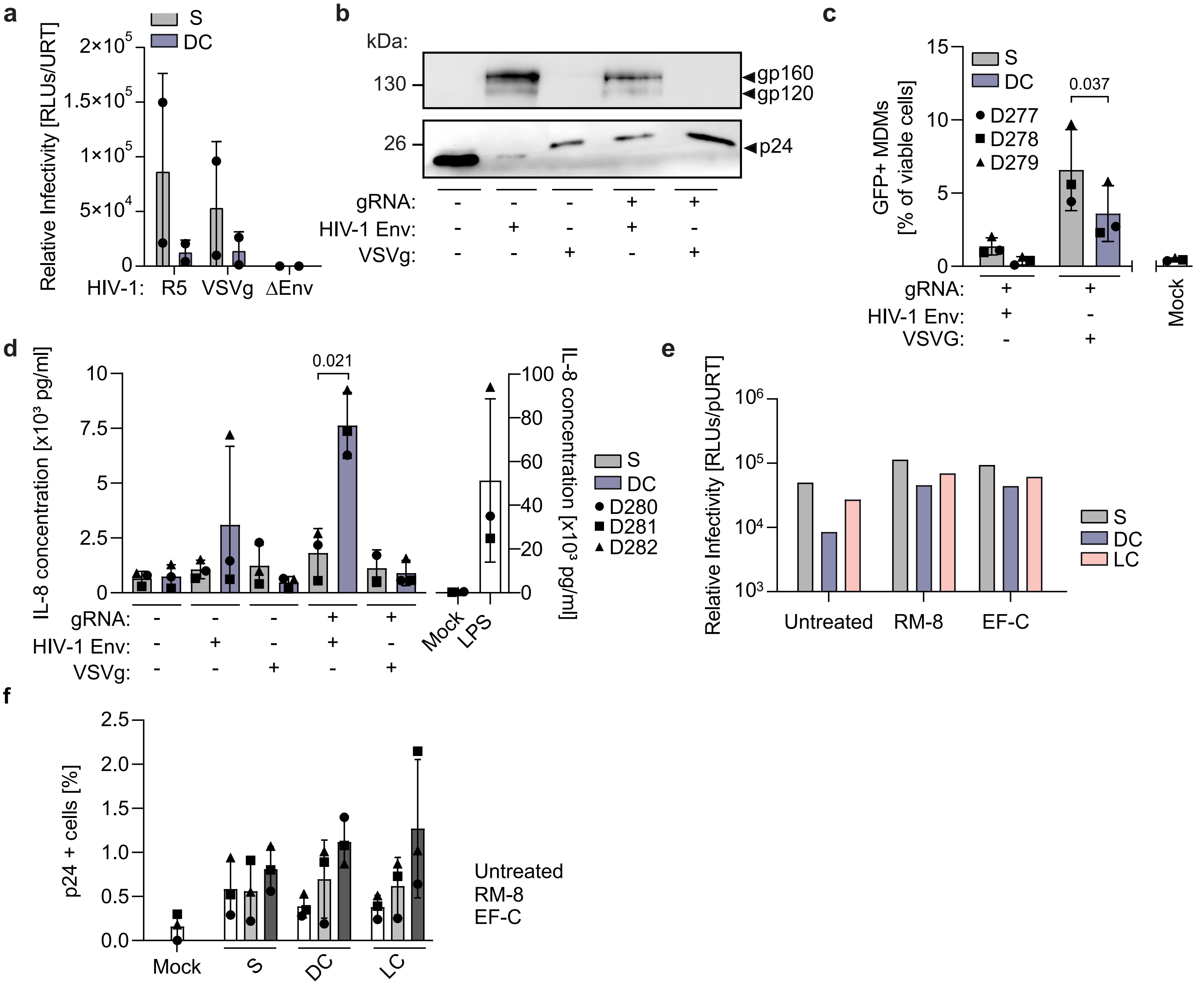

Supplementary Figure 4: The ERVI induced sensitization depends on the presence of HIV-1 Env and gRNA. **a**. The relative infectivity of S or DC derived HIV-1 NL4.3 R5, HIV1 ΔEnv VSVg or HIV-1 ΔEnv was determined after 16h of culture. **b**. Representative WB result of lentiviruses produced in the presence of HIV-1 Env, VSVg and/or a lentiviral gRNA. Efficient incorporation of p24 and HIV-1 Env was assessed by performing an SDS-PAGE followed by blotting and incubation with anti-p24 or anti-gp120 antibodies. **c**. Flow cytometry analysis of MDMs transduced with lentiviral vectors retrieved from suspension or collagen. Cells that were transduced with gRNA containing lentiviral particles produce GFP, which was detected by flow cytometry. **d**. Measurement of IL-8 release by ELISA from the supernatants of MDMs challenged with the different lentiviral particles. **e**. Relative infectivity determination. Equivalent amounts of S or DC derived HIV-1 NL4.3 R5 Vpr.Int.GFP virions were incubated with infectivity enhancers prior to infection of TZM-bl reporter cells for infectivity determination, revealing similar infectivity enhancement as shown for independent virus stocks in Fig. 2i. **f**. Flow cytometry quantification of the percentage of p24+ MDMs after challenge with virions as in a. Results represent the mean ± SD from one experiment (**e**), 2 experiments (**a**) or 3 independent donors (**c, d, e**). Significance is indicated by p-values, and was calculated by paired or unpaired t-tests (**c d, e**). Source data are provided as a Source Data file.

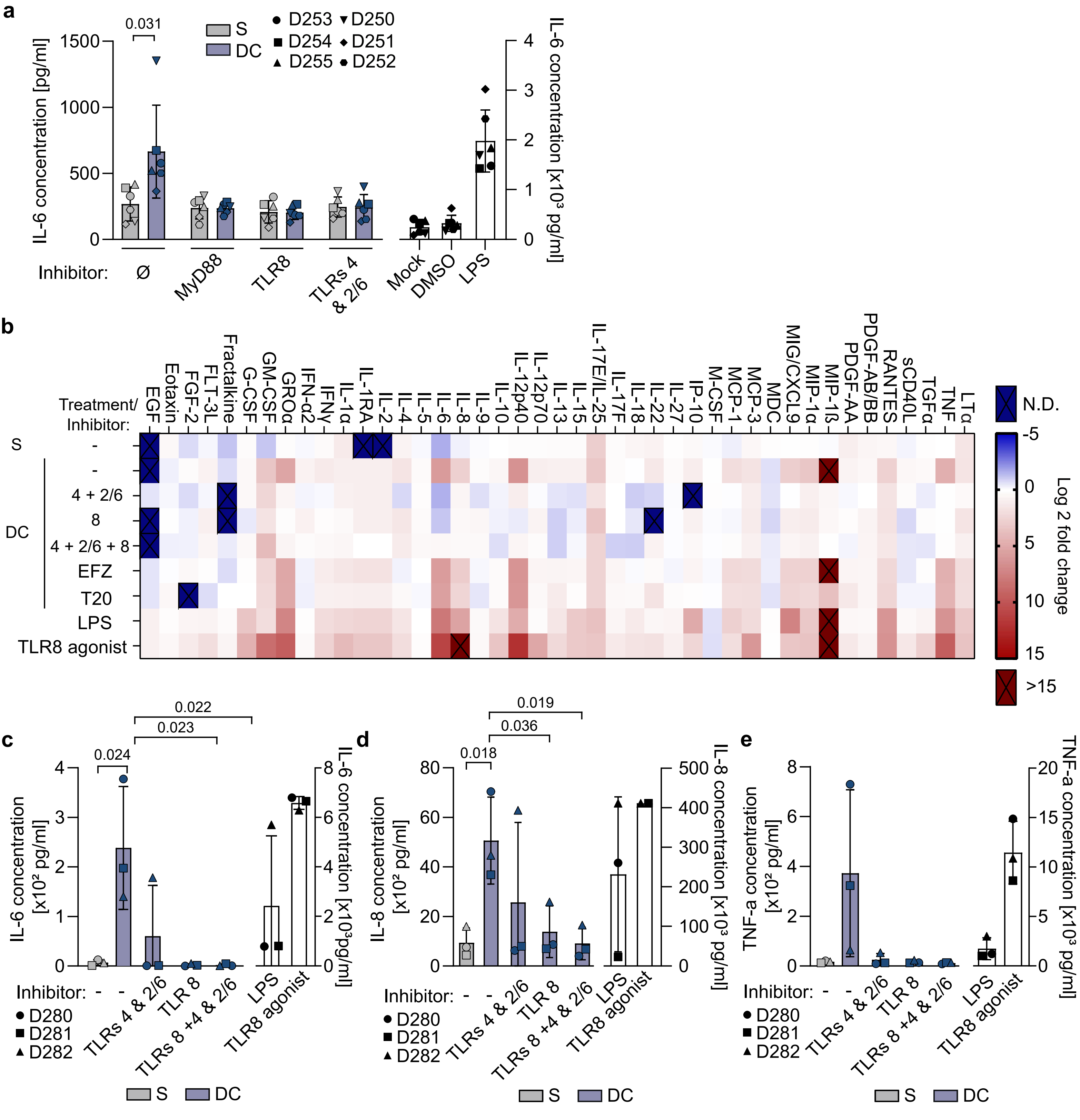

Supplementary Figure 5: TLRs 2 and 8 recognize collagen experienced particles to elicit a MyD88 dependent innate immune response. a. Measurement of IL-6 release by ELISA from the supernatants of MDMs challenged with S or DC derived HIV-1 NL4.3 R5 virions in presence or absence of different PRR inhibitors. **b**. Representative heat-map depicting the cytokine profiling for one donor, after challenge with virions from suspension or DC cultures, in presence or absence of HIV or PRR inhibitors. Dark blue & red colors indicate data points below and above the standard curve respectively. **c-e**. Quantification of the levels of IL-6 (**c**), IL-8 (**d**) or TNF (**e**) release for 3 donors from the cytokine profiling as in b. Results represent the mean ± SD from 3 (**c,d,e**) or 6 (**a**) independent donors. Significance is indicated by p-values, and was calculated by paired or unpaired t-tests. Source data are provided as a Source Data file.

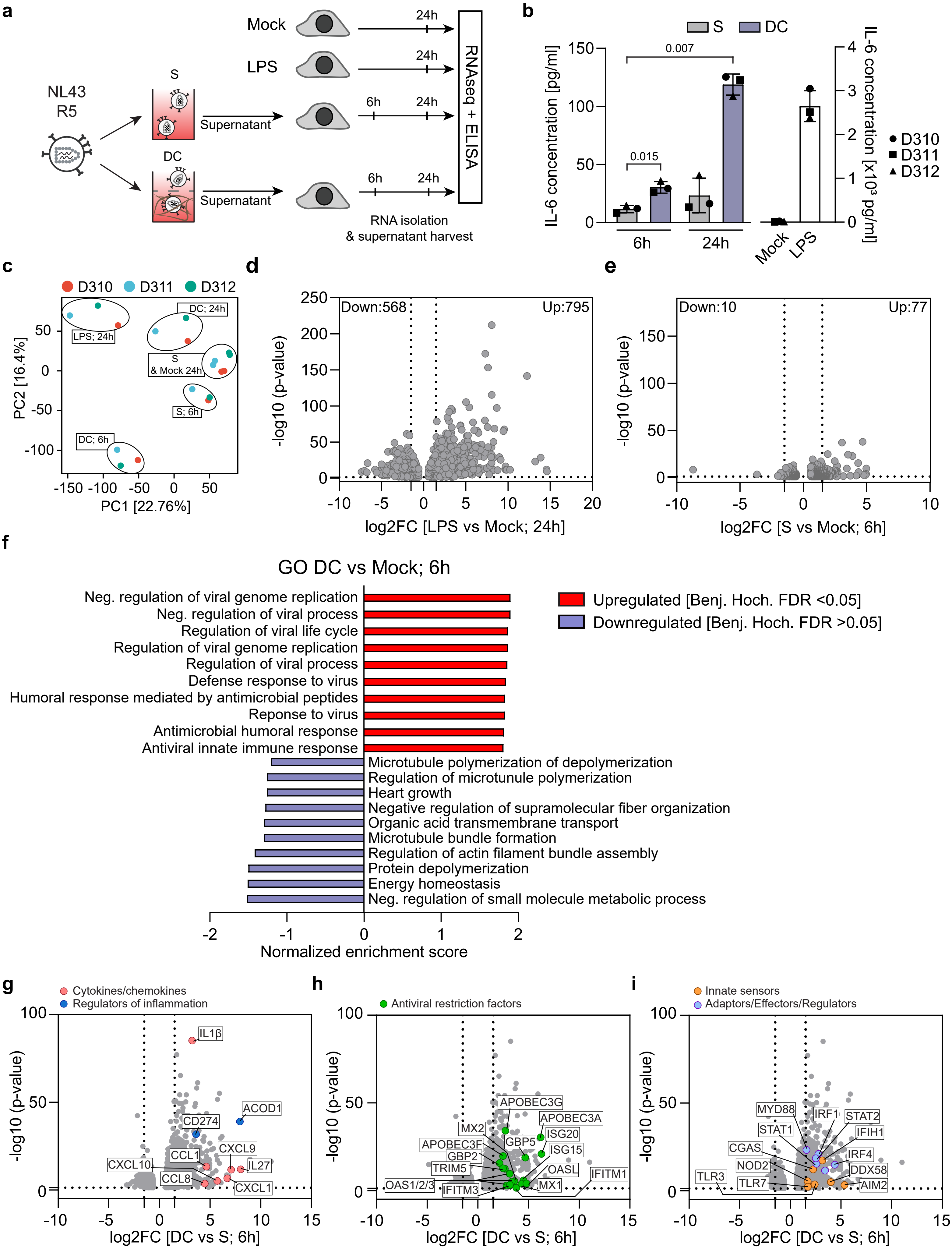
a
NL43 R5
S
DC
Mock
LPS
Supernatant
Supernatant
24h
6h
RNAseq + ELISA
RNA isolation
& supernatant harvest
b
S
DC
IL-6 concentration [pg/ml]
IL-6 concentration [x10³ pg/ml]
0.007
0.015
6h
24h
Mock
LPS
D310
D311
D312
c
D310
D311
D312
LPS; 24h
DC; 24h
S & Mock 24h
S; 6h
DC; 6h
PC2 [16.4%]
PC1 [22.76%]
d
Down:568
Up:795
-log10 (p-value)
log2FC [LPS vs Mock; 24h]
e
Down:10
Up:77
-log10 (p-value)
log2FC [S vs Mock; 6h]
f
GO DC vs Mock; 6h
Upregulated [Benj. Hoch. FDR <0.05]
Downregulated [Benj. Hoch. FDR >0.05]
Neg. regulation of viral genome replication
Neg. regulation of viral process
Regulation of viral life cycle
Regulation of viral genome replication
Regulation of viral process
Defense response to virus
Humoral response mediated by antimicrobial peptides
Reponse to virus
Antimicrobial humoral response
Antiviral innate immune response
Microtubule polymerization of depolymerization
Regulation of microtunule polymerization
Heart growth
Negative regulation of supramolecular fiber organization
Organic acid transmembrane transport
Microtubule bundle formation
Regulation of actin filament bundle assembly
Protein depolymerization
Energy homeostasis
Neg. regulation of small molecule metabolic process
Normalized enrichment score
g
Cytokines/chemokines
Regulators of inflammation
IL1β
ACOD1
CD274
CCL1
CXCL9
CXCL10
IL27
CCL8
CXCL1
-log10 (p-value)
log2FC [DC vs S; 6h]
h
Antiviral restriction factors
APOBEC3G
APOBEC3A
MX2
ISG20
GBP5
APOBEC3F
ISG15
GBP2
TRIM5
OASL
IFITM1
OAS1/2/3
MX1
IFITM3
-log10 (p-value)
log2FC [DC vs S; 6h]
i
Innate sensors
Adaptors/Effectors/Regulators
MYD88
IRF1
STAT2
STAT1
IFIH1
CGAS
IRF4
NOD2
DDX58
TLR3
TLR7
AIM2
-log10 (p-value)
log2FC [DC vs S; 6h]

Supplementary Figure 6: Transcriptome of MDMs at early time points after challenge with suspension or collagen primed HIV-1 NL4.3 R5 virus. a. Experimental workflow. MDMs from 3 donors were challenged with suspension or dense collagen primed virions for 6h or 24. As control, mock or LPS treated MDMs were harvested after 24h. The culture supernatants were harvested for IL-6 ELISA analysis, and RNA was isolated at the indicated time points for RNAseq. **b**. Measurement of IL-6 release by ELISA from the supernatants of MDMs challenged with suspension or collagen primed virions, or left untreated or incubated with for the indicated timepoints. **c**. Principal component analysis of RNA-seq data from the separate conditions. **d**. Volcano plot illustrating the differential gene expression between untouched macrophages (mock) and cells challenged with suspension virus for 6h. The number of down- and upregulated genes are indicated. **e**. Volcano plot illustrating the differential gene expression between untouched macrophages (mock) and LPS treated cells for 24h. The number of down- and upregulated genes are indicated. f. Gene ontology analysis of biological processes repressed or induced in MDMs challenged with collagen primed virions as compared to cells challenged with suspension virus for 6h. FDR is indicated (only upregulated pathways have a q-value <0.05). Top deregulated genes for each pathway are highlighted. **g-i.** Differential gene expression analysis. Volcano plots as in Fig. 6g, in which cytokine/chemokines or regulators of inflammation (**g**), antiviral restriction factors (**h**) or innate sensors, adaptors/effectors/regulators (**i**) are highlighted. Results represent the mean ± SD from 3 independent donors (**b,c,d,e**). Significance is indicated by p-values, and was calculated by one-way ANOVA, Geisser-Greenhouse correction, Tukey's post-test (**b**), Differential gene expression analysis was performed using DESeq2: genes with adjusted p-value <0.05 were considered significant (**d,e; g-i**), for GSEA analysis, p-values were corrected using Benjamini-Hochberg FDR (**f**) Source data are provided as a Source Data file.

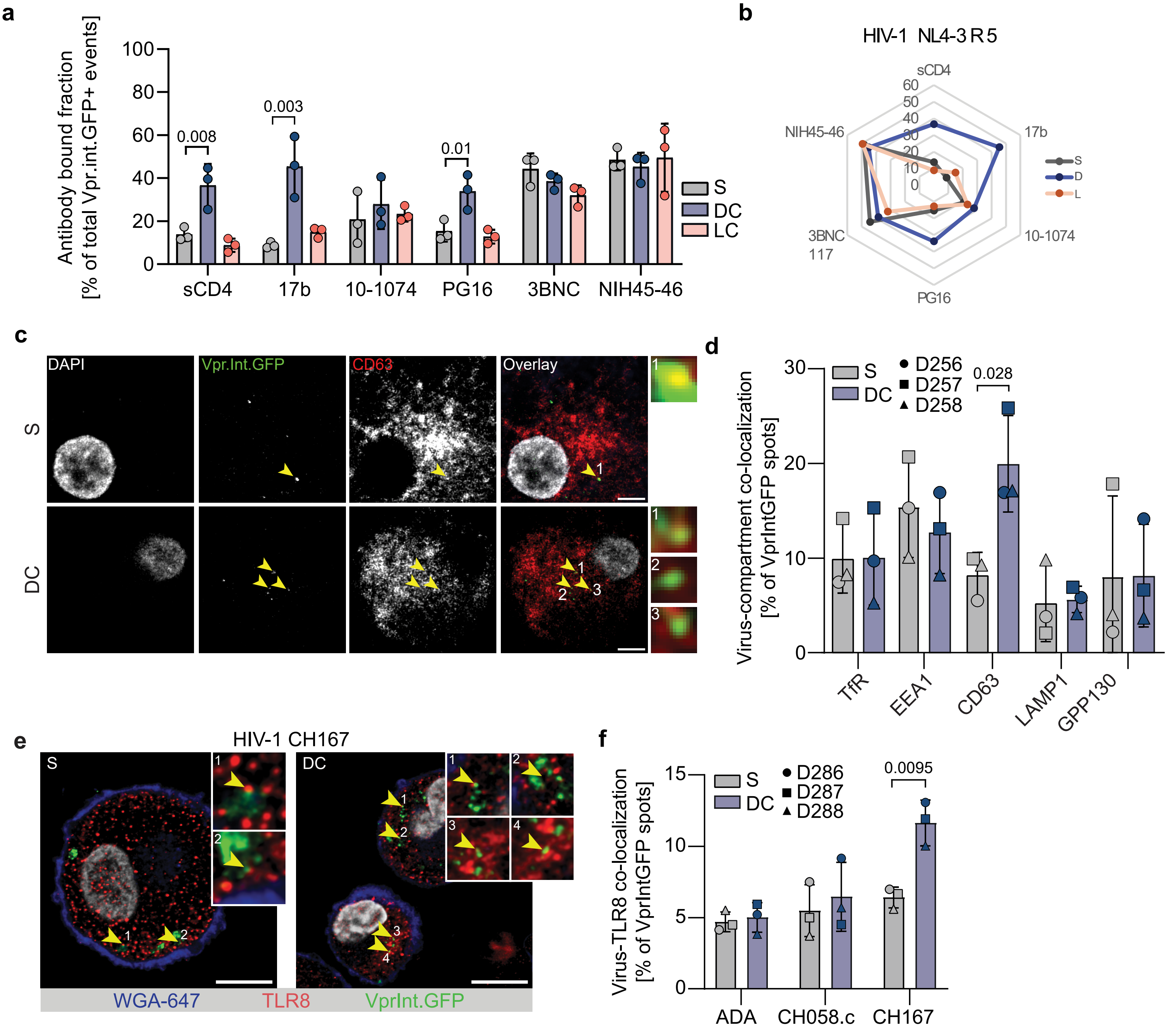

Supplementary Figure 7: HIV-1 Env is structurally rearranged after contact of virions with collagen fibers. a. Quantification of the frequency of antibody/sCD4 binding to differentially cultured HIV-1 NL4.3 R5 Vpr.Int.GFP virions as shown in Fig. 7c. **b**. Spider plot representing the frequency of antibody binding to HIV-1 NL4.3 R5 as in (a). **c**. Representative micrographs. MDMs were challenged for 3h with S or DC derived HIV-1 NL4.3 R5 Vpr.Int.GFP virions and immunostained for different compartments (CD63 shown). Yellow arrows: Vpr.Int.GFP/CD63+ colocalization. Scale bar: 5 µm. **d**. Quantification of Virus compartment colocalization from micrographs as in c. **e**. Representative micrographs of MDMs challenged for 3h with S or DC derived HIV-1 CH167 Vpr.Int.GFP. Yellow arrows indicate Vpr.Int.GFP/TLR8 colocalization. WGA-647 was used to stain the plasma membrane. Scale bar: 10 µm. **f**. Quantification of Vpr.Int.GFP/TLR8 colocalization for different viral strains as in (e). Results represent the mean ± SD from 3 independent experiments (**a,b**) or 3 independent donors (**c-f**). Significance is indicated by p-values, and was calculated by paired t-tests or Wilcoxon matched-pairs signed rank test. Source data are provided as a Source Data file.

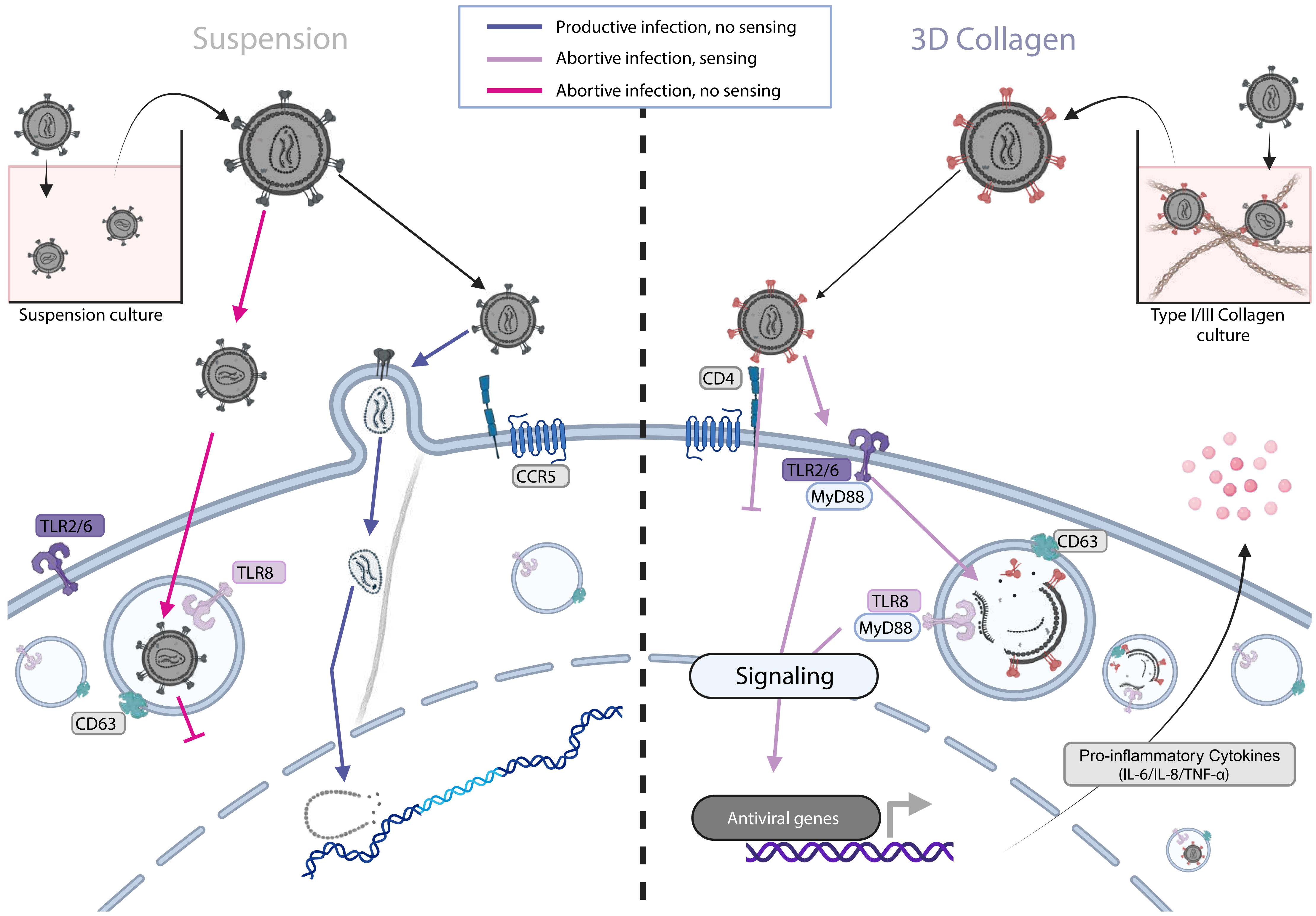

Suspension
Productive infection, no sensing
Abortive infection, sensing
Abortive infection, no sensing
3D Collagen
Suspension culture
Type I/III Collagen culture
CD4
CCR5
TLR2/6
MyD88
TLR8
CD63
Signaling
Antiviral genes
Pro-inflammatory Cytokines (IL-6/IL-8/TNF-α)

Supplementary Figure 8: Schematic model of the dual mechanisms by which ERVI impairs the infectivity of HIV-1 particles and sensitizes them for TLR2 and TLR8 innate immune recognition in MDMs. See discussion for details. Illustration created on biorender

FACS gating strategies:

1- Vpr-BlaM entry assay:

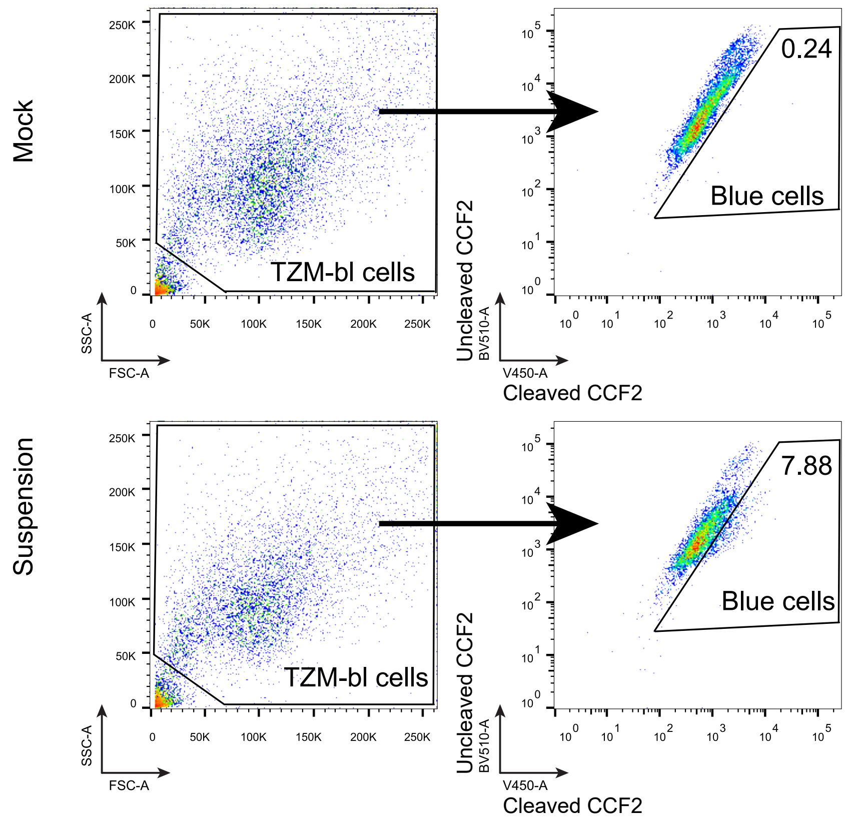

2- Frequency of p24+ cells (same strategy for MDMs or CD4+ T cells)

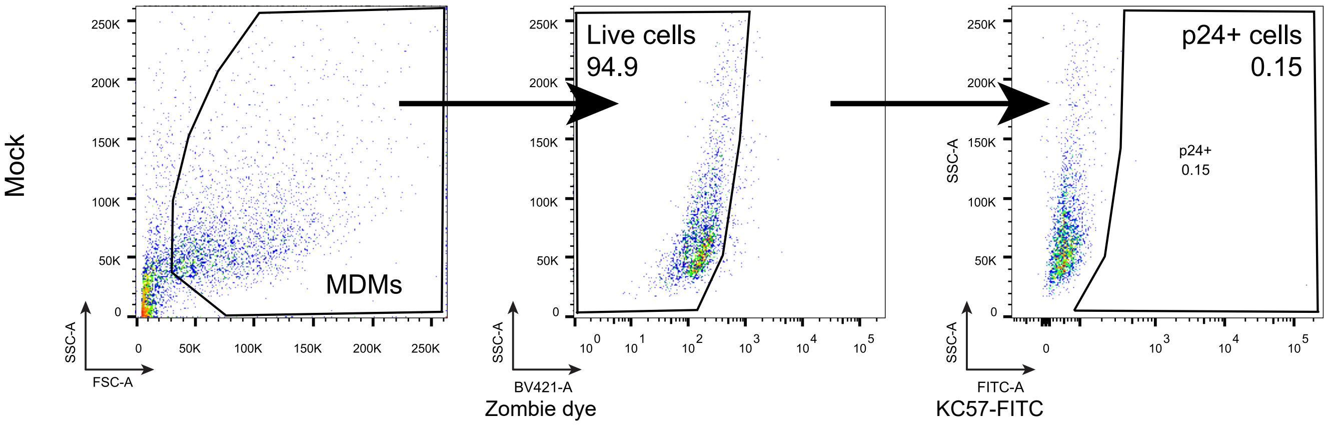

Supplementary Figure 9: Gating strategies employed in flow cytometry read outs.
Depiction of gating strategies employed to quantify viral entry in the Vpr-BlaM entry assays (see Fig.3) and for quantification of HIV-1 virus infectivity in primary cells (see Fig. 4).

References:

108. Bösl, K. et al. Coactivation of TLR2 and TLR8 in primary human monocytes triggers a distinct inflammatory signaling response.Front. Physiol. 9, 618 (2018).
109. Song, R. et al. IRF1 governs the differential interferon-stimulated gene responses in human monocytes and macrophages by regulating chromatin accessibility. Cell Rep. 34, 108891 (2021).
110. Teixeira-Coelho, M. et al. Differential post-transcriptional regulation of IL-10 by TLR2 and TLR4-activated macrophages. Eur. J. Immunol. 44, 856–866 (2014).
111. Oliveira-Nascimento, L., Massari, P. & Wetzler, L. M. The role of TLR2 in infection and immunity. Front. Immunol. 3, 79 (2012).
112. West, A. C. et al. Identification of a TLR2-regulated gene signatureassociated with tumor cell growth in gastric cancer. Oncogene 36, 5134–5144 (2017).
113. Hari, P. et al. The innate immune sensor Toll-like receptor 2 controls the senescence-associated secretory phenotype. Sci. Adv. 5, eaaw0254 (2019).
114. Coch, C. et al. Human TLR8 senses RNA from plasmodium falciparum-infected red blood cells which is uniquely required for the IFN-γ response in NK cells. Front. Immunol. 10, 371 (2019).
115. Saruta, M. et al. TLR8-mediated activation of human monocytes inhibits TL1A expression. Eur. J. Immunol. 39, 2195–2202 (2009).
116. Cervantes, J. L. et al. Phagosomal signaling by Borrelia burgdorferi in human monocytes involves Toll-like receptor (TLR) 2 and TLR8 cooperativity and TLR8-mediated induction of IFN-beta. Proc.Natl. Acad. Sci. USA 108, 3683–3688 (2011).
117. Breuer, K. et al. InnateDB: systems biology of innate immunity and beyond-recent updates and continuing curation. Nucleic Acids Res. 41, D1228–D1233 (2013).

References:

108. Bösl, K. et al. Coactivation of TLR2 and TLR8 in primary human monocytes triggers a distinct inflammatory signaling response.Front. Physiol. 9, 618 (2018).
109. Song, R. et al. IRF1 governs the differential interferon-stimulated gene responses in human monocytes and macrophages by regulating chromatin accessibility. Cell Rep. 34, 108891 (2021).
110. Teixeira-Coelho, M. et al. Differential post-transcriptional regulation of IL-10 by TLR2 and TLR4-activated macrophages. Eur. J. Immunol. 44, 856–866 (2014).
111. Oliveira-Nascimento, L., Massari, P. & Wetzler, L. M. The role of TLR2 in infection and immunity. Front. Immunol. 3, 79 (2012).
112. West, A. C. et al. Identification of a TLR2-regulated gene signatureassociated with tumor cell growth in gastric cancer. Oncogene 36, 5134–5144 (2017).
113. Hari, P. et al. The innate immune sensor Toll-like receptor 2 controls the senescence-associated secretory phenotype. Sci. Adv. 5, eaaw0254 (2019).
114. Coch, C. et al. Human TLR8 senses RNA from plasmodium falciparum-infected red blood cells which is uniquely required for the IFN-γ response in NK cells. Front. Immunol. 10, 371 (2019).
115. Saruta, M. et al. TLR8-mediated activation of human monocytes inhibits TL1A expression. Eur. J. Immunol. 39, 2195–2202 (2009).
116. Cervantes, J. L. et al. Phagosomal signaling by Borrelia burgdorferi in human monocytes involves Toll-like receptor (TLR) 2 and TLR8 cooperativity and TLR8-mediated induction of IFN-beta. Proc.Natl. Acad. Sci. USA 108, 3683–3688 (2011).
117. Breuer, K. et al. InnateDB: systems biology of innate immunity and beyond-recent updates and continuing curation. Nucleic Acids Res. 41, D1228–D1233 (2013).
