# Supplementary material for: A tissue-intrinsic mechanism sensitizes HIV-1 particles for TLR-triggered innate immune responses
Source: Nat Commun. 2026 May 9;17:4209. doi: 10.1038/s41467-026-72586-3 (PMC13157497; doi:10.1038/s41467-026-72586-3)
Supplement: Supplementary file 2 — Description of Additional Supplementary Files [file 41467_2026_72586_MOESM2_ESM.pdf]

Description of Additional Supplementary Files

File Name: Supplementary Data 1

Description: TLR2 gene signature enrichment in DC vs S; 6h.

Genes deregulated upon TLR2 activation are indicated as extracted from several reports in literature. The species, cell type and regulation context are listed. Data extracted from [108,109,110,111,112,113]. See references for statistical tests used to determine significance.

File Name: Supplementary Data 2

Description: TLR8 gene signature enrichment in DC vs S; 6h.

Genes deregulated upon TLR2 activation are indicated as extracted from several reports in literature. The species, cell type and regulation context are listed. Data extracted from [108,109,114,115]. See references for statistical tests used to determine significance.

File Name: Supplementary Data 3

Description: Genes co-regulated by TLR2 and TLR8 enrichment in DC vs S; 6h.

Genes co-regulated upon TLR2 and TLR8 activation are indicated as extracted from several reports in literature. The species, cell type and regulation context are listed. Data extracted from [108,109,116]. See references for statistical tests used to determine significance.

File Name: Supplementary Data 4

Description: Innate Immune Response genes enrichment in DC vs Mock; 6h.

The list of DEGs was compared to the curated InnateDB database (Innate Immunity Genes) [117]. Differential gene expression analysis was performed using DESeq2: genes with adjusted p-value <0.05 were considered significant.

File Name: Supplementary Data 5

Description: Differential gene expression of MDMs challenged with DC virions vs Mock for 6h.

Differential gene expression analysis was performed using DESeq2: genes with adjusted p-value <0.05 were considered significant.

File Name: Supplementary Data 6

Description: Differential gene expression of MDMs challenged with DC vs S virions for 6h.

Differential gene expression analysis was performed using DESeq2: genes with adjusted p-value <0.05 were considered significant.

File Name: Supplementary Movie 1

Description: Localization to TLR8 containing endosomes of virions derived from suspension cultures.

HIV-1 CH167 virions retrieved from suspension cultures were used to challenge MDMs for 3h to allow internalization. Samples were then processed for confocal microscopy analysis after TLR8 immunostaining, followed by 3D reconstruction and segmentation using Imaris software. Source data are provided as a Source Data file.

File Name: Supplementary Movie 2

Description: Localization to TLR8 containing endosomes of virions derived from dense collagen cultures.

HIV-1 CH167 virions retrieved from dense collagen cultures were used to challenge MDMs for 3h to allow internalization. Samples were then processed for confocal microscopy analysis after TLR8 immunostaining, followed by 3D reconstruction and segmentation using Imaris software. Source data are provided as a Source Data file.
