# Supplementary material for: A tissue-intrinsic mechanism sensitizes HIV-1 particles for TLR-triggered innate immune responses
Source: Nat Commun. 2026 May 9;17:4209. doi: 10.1038/s41467-026-72586-3 (PMC13157497; doi:10.1038/s41467-026-72586-3)
Supplement: Supplementary file 11 — Reporting Summary [file 41467_2026_72586_MOESM11_ESM.pdf]

Corresponding author(s): Fackler O T

Last updated by author(s): Mar 31, 2026

Reporting Summary

Nature Portfolio wishes to improve the reproducibility of the work that we publish. This form provides structure for consistency and transparency in reporting. For further information on Nature Portfolio policies, see our Editorial Policies and the Editorial Policy Checklist.

Statistics

For all statistical analyses, confirm that the following items are present in the figure legend, table legend, main text, or Methods section.

n/a	Confirmed	
☐	☒	The exact sample size (*n*) for each experimental group/condition, given as a discrete number and unit of measurement
☐	☒	A statement on whether measurements were taken from distinct samples or whether the same sample was measured repeatedly
☐	☒	The statistical test(s) used AND whether they are one- or two-sided<br>*Only common tests should be described solely by name; describe more complex techniques in the Methods section.*
☐	☒	A description of all covariates tested
☐	☒	A description of any assumptions or corrections, such as tests of normality and adjustment for multiple comparisons
☐	☒	A full description of the statistical parameters including central tendency (e.g. means) or other basic estimates (e.g. regression coefficient) AND variation (e.g. standard deviation) or associated estimates of uncertainty (e.g. confidence intervals)
☐	☒	For null hypothesis testing, the test statistic (e.g. *F*, *t*, *r*) with confidence intervals, effect sizes, degrees of freedom and *P* value noted<br>*Give P values as exact values whenever suitable.*
☒	☐	For Bayesian analysis, information on the choice of priors and Markov chain Monte Carlo settings
☒	☐	For hierarchical and complex designs, identification of the appropriate level for tests and full reporting of outcomes
☒	☐	Estimates of effect sizes (e.g. Cohen's *d*, Pearson's *r*), indicating how they were calculated

Our web collection on statistics for biologists contains articles on many of the points above.

Software and code

Policy information about availability of computer code

Data collection	BioRad CFX 96 Real Time PCR detector for SG PERT, TECAN infinite M200PRO for luciferase assay and ELISA, BD FACS Celesta for flow cytometry, Titan Kryos for cryo-EM, Leica SP8 for confocal microscopy, PerkinElmer for Spinning disk, VisiView for TIRF microscopy, Intas Chemostar ECL imager for Western blot, LI-COR Odyssey DLx imaging system for western blot
Data analysis	GraphPad Prism, Image analysis was performed using Image J, Imaris and Icyi, Flow cytometry data analysis with FlowJo, Biorad XT for SG PERT, GSEA software for gene set enrichment analysis, Affinity designer (v2.5) was used to generate illustrations

For manuscripts utilizing custom algorithms or software that are central to the research but not yet described in published literature, software must be made available to editors and reviewers. We strongly encourage code deposition in a community repository (e.g. GitHub). See the Nature Portfolio guidelines for submitting code & software for further information.

Data

Policy information about availability of data

All manuscripts must include a data availability statement. This statement should provide the following information, where applicable:

- Accession codes, unique identifiers, or web links for publicly available datasets
- A description of any restrictions on data availability
- For clinical datasets or third party data, please ensure that the statement adheres to our policy

All data supporting the findings of this study are available within the article and its supplementary files. Any additional requests for information can be directed to, and will be fulfilled by, the corresponding authors

Research involving human participants, their data, or biological material

Policy information about studies with human participants or human data. See also policy information about sex, gender (identity/presentation), and sexual orientation and race, ethnicity and racism.

Reporting on sex and gender	Buffy coats were obtained from healthy donors without discrimination according to sex/gender.
Reporting on race, ethnicity, or other socially relevant groupings	Race, ethnicity, or other social grouping were not considered in the study design. Blood was obtained from the local German Red Cross.
Population characteristics	Human samples were obtained through the German Red Cross, harvested in the land Baden-Württemberg.
Recruitment	No patients were recruited specifically for this study. No bias expected to affect this study
Ethics oversight	All procedures performed in this study were approved by the Ethics committee

Note that full information on the approval of the study protocol must also be provided in the manuscript.

Field-specific reporting

Please select the one below that is the best fit for your research. If you are not sure, read the appropriate sections before making your selection.

☒ Life sciences ☐ Behavioural & social sciences ☐ Ecological, evolutionary & environmental sciences

For a reference copy of the document with all sections, see nature.com/documents/nr-reporting-summary-flat.pdf

Life sciences study design

All studies must disclose on these points even when the disclosure is negative.

Sample size	No statistical method was used to predetermine sample sizes used in this paper. Sample sizes for each experiment are indicated in the figure legends.
Data exclusions	No data was excluded.
Replication	All experiments were independently performed using at least 3 biological replicates.
Randomization	The samples used in this study were all divided randomly in separate gorups.
Blinding	Data was not blinded for analysis

Reporting for specific materials, systems and methods

We require information from authors about some types of materials, experimental systems and methods used in many studies. Here, indicate whether each material, system or method listed is relevant to your study. If you are not sure if a list item applies to your research, read the appropriate section before selecting a response.

Materials & experimental systems

n/a	Involved in the study
[ ]	[x] Antibodies
[ ]	[x] Eukaryotic cell lines
[x]	[ ] Palaeontology and archaeology
[x]	[ ] Animals and other organisms
[x]	[ ] Clinical data
[x]	[ ] Dual use research of concern
[x]	[ ] Plants

Methods

n/a	Involved in the study
[x]	[ ] ChIP-seq
[ ]	[x] Flow cytometry
[x]	[ ] MRI-based neuroimaging

Antibodies

Antibodies used

Flow cytometry:
anti-p24 KC57-FITC (1:100,#6604665, Beckman Coulter)
Zombie Violet Fixable Viability dye (1:1000, 423113, Biolegend)

Western Blotting:
rabbit polyclonal antiserum against HIV-1 gp120 was kindly provided by Valerie Bosch (DKFZ, Heidelberg, Germany) (used 1:1000)
rabbit polyclonal antiserum against HIV-1 p24 (1:1000, Kindly provided by Prof. Dr. Barbara Müller, CIID, Heidelberg)
Goat anti-rabbit IgG-HRP (1:1000, 65-6120, Invitrogen)
Goat anti-rabbit IRDye700/800 conjugated antibodies (1:20000, 611-1302, Rockland)

Immunofluorescence:
mouse anti-TLR8 (1:200, 67317, Proteintech)
mouse anti-human EEA1 (1:200, 68065, Proteintech)
mouse anti-TfR (1:250, 13-6800, Invitrogen)
sheep anti-TGN46 (1:100, AHP500GT, BioRad)
mouse anti-CD63 (1:200, 556019, BD)
mouse anti-TfR (1:250, 13-6800, Invitrogen)
rabbit anti-GPP130 (1:100, 923801, Biolegend)
mouse anti-LAMP1 (1:50, ab25630, Abcam)
anti-HIV-1 gp120 (PG16. 15 ug/ul, BEI resources)
anti-HIV-1 gp120 (3BNC117 15 ug/ul, BEI resources)
anti-HIV-1 gp120 (10-1074, 15 ug/ul, BEI resources)
anti-HIV-1 gp120 (NIH45-46, 15 ug/ul, BEI resources)
anti-human-gp120 (17b, 15 ug/ul, BEI resources)

List the secondary antibodies:
goat anti-mouse IgG1 Alexa Fluor 568(1:200, Invitrogen)
goat anti-human IgG1 Alexa Fluor 568 (1:200, Invitrogen)
goat anti-sheep IgG1 Alexa Fluor 568 (1:200, Invitrogen)

Validation

We did not validate the antibodies ourselves. When possible, we selected antibodies that were validated by KO by the manufacturer

Eukaryotic cell lines

Policy information about cell lines and Sex and Gender in Research

Cell line source(s)	HEK293T, Huh 7.5, and TZM-bl cells used in this study were obtained from:
Authentication	Cell lines were not authenticated by ourselves.
Mycoplasma contamination	All cell lines were routinely tested for mycoplasma and no contamination was revealed.
Commonly misidentified lines (See ICLAC register)	No commonly misidentified cell line was used.

Plants

Seed stocks	Not applicable
Novel plant genotypes	Not applicable
Authentication	Not applicable

Flow Cytometry

Plots

Confirm that:

- [x] The axis labels state the marker and fluorochrome used (e.g. CD4-FITC).
- [x] The axis scales are clearly visible. Include numbers along axes only for bottom left plot of group (a 'group' is an analysis of identical markers).
- [x] All plots are contour plots with outliers or pseudocolor plots.
- [x] A numerical value for number of cells or percentage (with statistics) is provided.

Methodology

Sample preparation	Sample preparation of MDMs for flow cytometry is described in Methods section
Instrument	BD FACS Celesta
Software	Data was collected with the FACSuite (BD) and analysed using the FlowJo Software (FlowJo LLC)
Cell population abundance	No sorting was performed, pure population of detached MDMs were analysed to determine viability and p24 positivity.
Gating strategy	Gating on cells by FSC/SSC, homogeneous distribution, gating on clear population of MDMs. Dead cells were excluded (negative for Zombie Violet dye). Within the live cell population, p24 positive cells (positive for KC57-FITC antibody) were gated on in the FITC channel (gating positioned as compared to uninfected cells stained with KC57-FITC).

- [x] Tick this box to confirm that a figure exemplifying the gating strategy is provided in the Supplementary Information.
